# Antimalarial Drug Predictions Using Molecular Descriptors and Machine Learning against Plasmodium Falciparum

**DOI:** 10.3390/biom11121750

**Published:** 2021-11-24

**Authors:** Medard Edmund Mswahili, Gati Lother Martin, Jiyoung Woo, Guang J. Choi, Young-Seob Jeong

**Affiliations:** 1 Department of ICT Convergence, Soonchunhyang University, Asan-si 31538, Korea; medardedmund25@sch.ac.kr (M.E.M.); gatimartin@sch.ac.kr (G.L.M.); 2 Department of Pharmaceutical Engineering, Soonchunhyang University, Asan-si 31538, Korea; guangchoi@sch.ac.kr; 3 Department of Computer Engineering, Chungbuk National University, Cheongju 28644, Korea

**Keywords:** antimalarial drug, machine learning, plasmodium falciparum, molecular descriptor, drug discovery, feature selection, PaDEL

## Abstract

Malaria remains by far one of the most threatening and dangerous illnesses caused by the plasmodium falciparum parasite. Chloroquine (CQ) and first-line artemisinin-based combination treatment (ACT) have long been the drug of choice for the treatment and controlling of malaria; however, the emergence of CQ-resistant and artemisinin resistance parasites is now present in most areas where malaria is endemic. In this work, we developed five machine learning models to predict antimalarial bioactivities of a drug against plasmodium falciparum from the features (i.e., molecular descriptors values) obtained from PaDEL software from SMILES of compounds and compare the machine learning models by experiments with our collected data of 4794 instances. As a consequence, we found that three models amongst the five, namely artificial neural network (ANN), extreme gradient boost (XGB), and random forest (RF), outperform the others in terms of accuracy while observing that, using roughly a quarter of the promising descriptors picked by the feature selection algorithm, the five models achieved equivalent and comparable performance. Nevertheless, the contribution of all molecular descriptors in the models was investigated through the comparison of their rank values by the feature selection algorithm and found that the most potent and relevant descriptors which come from the ‘Autocorrelation’ module contributed more while the ‘Atom type electrotopological state’ contributed the least to the model.

## 1. Introduction

Regardless of the fact that COVID-19 is by far the most serious current threat tragedy known as a global pandemic with hundreds of millions confirmed cases of COVID-19, including millions deaths, reported to the World Health Organization (WHO) in 2021, still approximately millions of people, especially Africans, died of malaria, tuberculosis, and HIV-related illnesses. These three diseases can be prevented or treated with timely access to appropriate and affordable medicines, vaccines, and other health services. However, less than 2% of drugs consumed in Africa are produced on the continent, meaning that a huge number of sick patients do not have access to locally produced drugs and may not afford to buy the imported ones. Without reliable access to medicines, more people, especially in Africa and a few parts of Asia, are susceptible to the three big killer diseases on their respective continents. Globally, 50% of children under five who die of pneumonia, diarrhea, measles, HIV, tuberculosis, and malaria are in Africa, according to the WHO. Although the organization continues to struggle with making medicine more conveniently, in order to be accessible, such as having medicines be continuously available and inexpensive at designated and authorized health facilities located within a reasonable distance of the people, malaria remains by far the most threatening and dangerous illness due to its profoundly negative impact and detrimental influence on global communities in terms of social, political, and economical growth particularly in developing countries [1,2].

Malaria is a life-threatening disease caused by plasmodium parasites that are transmitted to people through the bites of infected female anopheles mosquitoes, called malaria vectors. There are five well known existing parasite species that cause malaria in humans according to [3], and plasmodium falciparum among them is known to cause the most severe form of the disease whereby those who contract this form of malaria have a higher risk of death, so the majority of deaths due to malaria are caused by the plasmodium falciparum [4,5,6,7], and it is susceptible to naturally acquired host immunity. Notably the main burden of Malaria disease falls on young children [7]. Despite the organization’s current elimination struggle, which includes taking into account all possible controllable measures, the effectiveness of malaria prevention, control, and treatment is dependent on the sustained clinical efficacy of first-line artemisinin-based combination treatment (ACT), which is constantly threatened by the establishment of emergence and spread of drug resistance [8,9].

Chloroquine (CQ) has long been the drug of choice for the treatment of malaria; however, CQ-resistant parasites are now present in most areas where malaria is endemic [10,11]. Moreover, recent alarming reports observed the emergence of artemisinin-resistant parasites in Southeast Asia [12,13], which could derail the current elimination/eradication efforts, and again foster an increase in malaria cases and deaths [14,15,16]. Observation of this study indicated the emergence of artemisinin resistance of Plasmodium falciparum not only in Southeast Asia but also in Sub-Saharan Africa, Tanzania being the case of study [17]. Resistance has emerged to all classes of antimalarial drugs which have lost their clinical effectiveness [11,18,19,20,21]. Resistance to these gold standard drugs represents a serious threat for malaria eradication, which causes a tremendous increase in the number of deaths annually, with excess medical costs and productivity losses of about 146 and 385 million US$ per year, respectively [15,22]. In addition, drug discovery and development are extremely long (time-consuming), costly (expensive), complex due to the challenges and obstacles that emerge during the drug development process, an outrageous failure that led to enormous financial damage, and an inefficient process that typically costs about 2.6 billion US dollars and takes an average of 10 to 15 years from essential pre-clinical testing to market approval, remarkably clinical trials being by far the most expensive factor during the development process [23].

To tackle the task of drug discovery, various approaches have been proposed. Quantitative structure–activity relationship (QSAR) is a computational or mathematical modeling method to reveal relationships between physicochemical properties of chemical substances and their biological activities to obtain a reliable statistical model for the prediction of the activities of new chemical entities. The underlying principle is that variations in structural properties cause different biological activities [24], where structural properties refer to physico-chemical properties, and biological activities correspond to pharmacokinetic properties such as absorption, distribution, metabolism, excretion, and toxicity. High-throughput screening (HTS) is another scientific experimentation approach especially used in drug discovery that involves the use of automated equipment to rapidly test thousands to millions of samples for biological activity at the model organism, cellular, pathway, or molecular level for identifying potential drug candidates [25,26,27]. QSAR modeling is an essential, paramount tool, and an alternative method that can assist in the selection of lead molecules by using the information from reference active and inactive compounds during the model implementation and development for drug discovery process, since the screening of chemical libraries with traditional methods, such as HTS, is expensive and time consuming [28].

Machine learning (ML) models have emerged in recent years as a promising and potentially appropriate tool for data-driven predictions in pharmaceutical science research, such as quantitative structure–activity/property relationships (QSAR/QSPR), drug–drug interactions, drug repurposing, and pharmacogenomics [29]; hence, certainly, the drug discovery area is undoubtedly one of the sectors that will profit greatly and tremendously gain benefits from the success of ML [30]. For example, Ref. [31] addressed the major crucial and critical fundamental problems (i.e., poor solubility, bioavailability, and efficacy of drugs) that hinder the drug development process through improving specific physicochemical and biopharmaceutical properties of active pharmaceutical ingredients (APIs), by applying ML models to predict which pair of API and coformer will successfully result in the new cocrystal formation that eventually becomes new drug and medicine after the Food and Drug Administration (FDA) approval, from a set of chemical experiments between API and the coformer since the essential and difficult phase in cocrystal production as an auxiliary state-of-the-art form to boost and enhance drug development is the screening of suitable coformers for an API. Danishuddin et al. [9] established the development and rigorous validation of antimalarial predictive models using machine learning approaches and ultimately achieved an accuracy of ∼85.00%. Egieyeh et al. [6] achieved an accuracy of 85.94% with the support vector machine (SVM), where the dataset was a combination of molecular descriptors and fingerprints of natural products with antiplasmodial activity (NAA). Liu et al. [32] used general regression neural networks (GRNN) for predicting the antimalarial activity against plasmodium falciparum, and achieved the accuracy of 88.90%. They inherited the work of [9] notably, the only difference being the number of features (i.e., molecular descriptors). The aforementioned studies have shown successful findings, but they all have a common flaw: they only compared model performance such as accuracy without meticulously looking at feature relevance.

This study focused on the development of machine learning models for predicting anti-malaria drugs. The problem is basically a binary classification on two labels (e.g., ‘active’, ‘inactive’), and we use the dataset of anti-malaria activity against plasmodium falciparum. To generate feature vectors, we use PaDEL-Descriptor software [33], one of the widely-used descriptor calculators that calculates molecular descriptors (MD) and fingerprints; it extracts descriptor values from simplified molecular-input line-entry system (SMILES) strings of the verified experimental anti-malaria drug compounds that were converted from two databases: ChEMBL database [34] and PubChem database [35].

The contributions of this paper can be summarized as follows. To begin, we not only extract descriptor values for compounds, but also analyze and investigate which descriptors are more significant, demonstrating that we can achieve decent results even if only a tiny subset of the descriptors are used. Following that, we conduct experiments to compare ML models and discover that three amongst the implemented models achieved equivalent results (i.e., comparable performance). The last but not least, we make our dataset available online via the website (https://sites.google.com/view/medardemswahili/ (accessed on 8 August 2021)) in the hopes of assisting many other researchers, as a benchmark to easily develop improved models.

## 2. Materials and Methods

**Figure 1:**
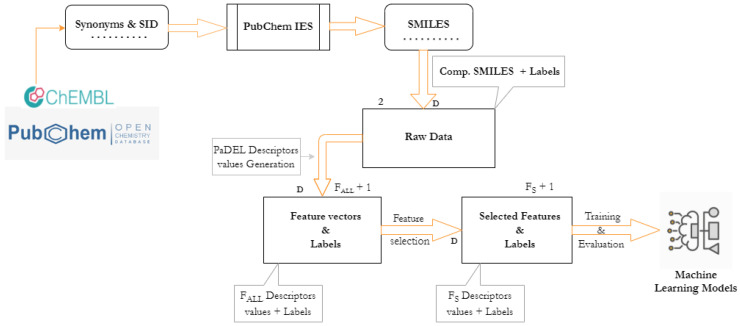
The development process for antimalarial drug prediction, from data gathering through ML models deployment.

We effectively tackle a binary classification problem by building ML models to predict a label (e.g., “active” or “inactive”) for a given experimentally verified antimalarial drug candidate from public chemical databases. The class label ‘active’ implies that the drug candidate compounds would successfully react against plasmodium falciparum parasite species, while the label ‘inactive’, there would be no reaction against plasmodium falciparum parasite species. Firstly, we obtain attributes (i.e., features) of the experimental antimalarial drug candidates compounds as depicted in Figure 1, from SMILES that were derived from their respective synonyms and Substance IDs (SID). Then, using feature selection algorithms, we choose some promising features, which are fed into the models that discover patterns behind the drug candidates’ compounds.

### 2.1. Materials

#### 2.1.1. Data

The verified antimalarial drug candidate compounds were downloaded from public chemical databases ChEMBL [34] and PubChem [35] in synonyms and SID format. We converted them into their respective and appropriate SMILES using the PubChem Identifier Exchange Service [36] as depicted in Figure 1.

The classification of active and inactive was done according to the antiplasmodial activities of the compounds with $IC_{50}$ of 10μM as a threshold. In general, compounds having an ($IC_{50}\leq 10\;\mu$M) will likely be ’active,’ implying that there will be a high number of active molecules. However, no experimental platform could possibly produce such a high percentage of active molecules [9]. As a result, the best model should discover molecules with an affinity >10μM in order to make the most of expensive experimental validation. The decision boundary for active compounds was determined at $IC_{50}\leq 1\;\mu$M [9]. The compound with ($IC_{50}\leq 1\;\mu$M) were set as ‘active’ and ‘inactive’ ($IC_{50}$: > 1 μM). The active instances are experimentally verified as active antimalarial drug candidates, whereas the inactive instances are experimentally verified as unsuccessful candidates. After filtering out some duplicated records out, we got a total of 4794 antimalarial drug candidate compounds, where it consists of 2070 and 2724 instances for active and inactive classes, respectively. The dataset is an |D| × 4 matrix, where |D| is the number of total instances. We converted the labels into a numerical form (i.e., ‘active’ = 1, and ‘inactive’ = 0) shown in Table 1 as a few samples. As the SMILES (e.g., ‘Canonical_Isomeric_SMILES’ in Table 1) is just a text, it is converted into real-numbered feature vectors using a certain calculator before it is fed into the models.

#### 2.1.2. Molecular Descriptors

Quantitative structure–property relationships (QSPR) models are frequently developed using molecular descriptors, and PaDEL is amongst the attractive and well-known tools to extract descriptors [33]. There are various tools used in cheminformatics [31] such as Mordred [37], PyDPI [38], Rcpi [39], Dragon [40], and cinfony [41], which is a collection or a wrapper of other libraries such as Open Babel [42], RDKit [31] (http://www.rdkit.org (accesssed on 22 June 2021)), and Chemistry Development Kit (CDK) [43]. We decided to utilize PaDEL because of its advantages: it provides approximately 1875 molecular descriptors within a brief execution time, and it is simple to install and utilize. The process of generating molecular descriptors is as follows: first, we prepare canonical and isomeric SMILES strings for each compound of antimalarial drug that are downloadable from PubChem Identifier Exchange Service. Second, we use the selected tool to obtain the features, as shown in the middle in Figure 1. Thereafter, obtaining a $F_{ALL}$ dimensional real-numbered feature vector from each antimalarial compound, we add a label column that resulted in a *D* feature vectors of $F_{ALL}+1$ dimension. Notably, the only molecular descriptors obtained and used in this study were 1D and 2D descriptors, and the $F_{ALL}$ = 1444.

### 2.2. Methods

As the dataset shown in Table 2 is balanced, we performed 10-fold cross validation while maintaining the balanced ratio; for each cross validation, we had around 4314 and 480 instances for training and testing, respectively. We denote the size of training dataset as $|D_{train}|$, and the size of test dataset as $|D_{test}|$, where |D|=$|D_{train}|$+$|D_{test}|$. We employ averaged accuracy, precision, recall, and F1 scores throughout all experimental findings.

Before passing the $|D_{train}|\times F_{ALL}+1$ real-numbered matrix to machine learning models, we scale or standardize the feature values in our data using both scaling methods (i.e., standardization and normalization) and then compared the results of both standardized and normalized data using ANN. Ultimately, the performance obtained when utilizing standardized data was superior to that obtained when using normalized data. Only training data are used in this process; the mean μ and standard deviation σ are derived using just the training data. We used scikit-learn [44,45] to implement the standardization because we discovered that it is superior to normalization (i.e., 0–1 values scaling) for our dataset. ML models are designed to give labels $y\in {\left\{0,1\right\}}^{|D_{train}|}$ where ‘active’ = 1 and ‘inactive’ = 0, based on the standardized matrix $X\in R^{|D_{train}|\times F_{ALL}}$.

We have implemented various ML models such as artificial neural network (ANN), support vector machine (SVM) [46], random forest (RF) [47], extreme gradient boost (XGB) [48], and Logistic Regression (LR) [49]. The ANN is recognized to be useful in a variety of research fields, including image analysis, natural language processing, and speech recognition; if it has a deep structure, it is a deep learning model (i.e., multiple hidden layers) [31]. The SVM is known to be successful in many classification applications and tasks [50], and it identifies a decision boundary based on boundary examples or instances (i.e., support vectors). The RF and XGB are both standard and common ensemble techniques, although the RF employs a bagging strategy while the XGB uses a boosting strategy [31]. The LR, a model with the sigmoid function often utilized by statisticians to describe properties of population growth in ecology, is rising quickly and maxing out at the carrying capacity of the environment.

Although there have been research studies that used molecular descriptors as features to train ML models [6,9], most of these studies simply provided the descriptors to the models without doing a critical and essential analysis of the descriptors. It is obvious that the performance of ML models strongly depends on the feature definition; wisely chosen molecular descriptors as features may give good performance even if we utilize a much smaller number of features. In this study, feature selection methods are employed to determine the importance of descriptors and then we use a group of promising and potential ones that we discovered.

We denote the number of selected features as $F_{S}$ as illustrated in the middle of Figure 1. Two feature selection algorithms are employed: Recursive Feature Elimination (RFE) and K-best algorithm. The K-best is a filter-based algorithm that selects potential features according to a particular function σ(f,c), where *f* and *c* are a feature and a label, respectively, while the RFE is a wrapper-based algorithm that treats the feature selection as a search problem [31], and eliminates unpromising features on a regular basis until only the desired number of features remains. The ANN model was used as an estimator of the RFE algorithm and took the ANOVA F-value as the function σ.

## 3. Results

Before we compare several well-known ML models by experimental results, we firstly compare and find the promising feature selection algorithm. The comparison will be fair only if we use the same features for all models; the models are compared with the same features chosen by the best feature selection algorithm.

### 3.1. Feature Selection Algorithms

**Figure 2:**
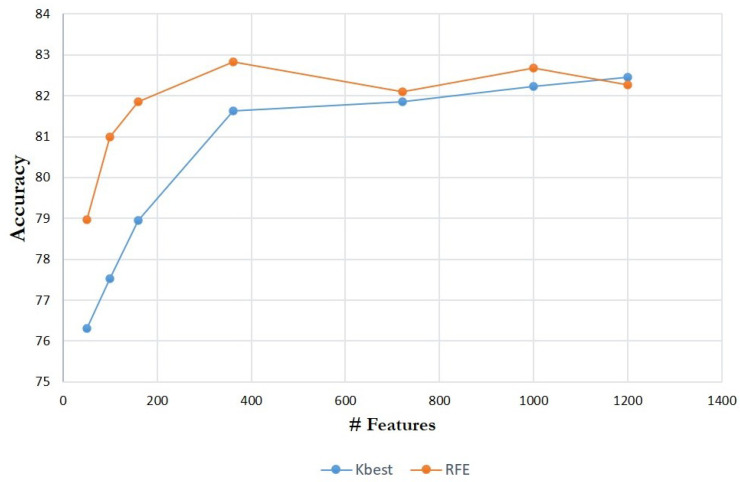
Averaged test set accuracy comparison using feature selection algorithms, against the number of $F_{S}$.

Through averaged test set accuracy with the number of features $F_{S}$ varying, the two feature selection algorithms (i.e., RFE and K-best) were examined and compared. The results are shown in Figure 2 with $F_{S}$ ranging from 50 to 1200, and the classifier employed here is ANN. With greater $F_{S}$, the K-best algorithm seems generally to have slightly greater accuracy than the RFE approach; otherwise, RFE performs better. As a result, we may say that the RFE algorithm is preferable if we seek efficiency (e.g., fewer parameters). In terms of feature dimension, because its dimension is merely a fifth of the total and its precision is equivalent in terms of accuracy, $F_{S}=$ 300∼400 may be a viable choice.

### 3.2. Model Comparison

We merged the datasets after downloading them from the aforementioned public databases, resulting in a single dataset *D* where |D| = 4794. Some machine-learning models (e.g., artificial neural networks with random initialization) are known to behave differently even if they are trained using the same dataset, so we randomly shuffled all instances of *D* and obtained five different datasets having the same size of |D|. Specifically, during shuffling, all criteria were taken into account to avoid data linking by ensuring that the total number of instances and features remained the same by keeping track of all the steps performed. All experimental results are averaged across the five datasets. Following that, we performed 10-fold cross validation for each dataset, and computed averaged test set accuracy, precision, recall, and F1 scores. A grid search employing a wee portion (e.g., 10%) of the training set as a validation set is used to find the optimal parameter settings for ML models.

The summarization of the parameter settings is as shown in Table 3. The ANN has two hidden layers of 100 nodes since we observed that it performs better than other complex structures with numerous layers and nodes, all of which were tested using the same standardized data; the reason for this could be the little and limited quantity of the dataset, which could lead to an over-fitting problem due to the high complexity of the model.

Table 4 below summarizes the test set accuracy of ML models. It is worth noting that the comparison of experimental outcomes of the models is the main focus of this section, not the feature selection techniques. The accuracy values are calculated by averaging the aforementioned independent datasets’ results. The XGB delivers the finest accuracy (e.g., 0.8303) amongst the implemented models, but the RF performed better with the number of features ≤ 160. The ANN and RF are comparable to the XGB, and it is the best when $F_{S}$ = 361 and $F_{S}$ = 1000. Because models function faster when feature dimensions are tiny, the XGB and RF may be preferable if we desire more efficiency without sacrificing or losing much accuracy.

One could argue that, if the model’s sensitivity is not great enough, it is useless. Table 5 and Table 6 are per-label test set precision and recall, respectively. The XGB gives the finest test set recall of ‘success’ label (e.g., 0.8068) without precision being greatly lost (e.g., 0.8477) followed by ANN when considering $F_{S}$ = 361 since all models in one way or the other performed remarkably better with this set of features. In terms of the precision, the RF appears the best, with a successful precision (i.e., ‘active’ label) of 0.8583, while the ANN and XGB may be preferred if we want to find as many potential chemical compound candidates as possible.

Table 7 shows the test set F1 scores for each label, and the ANN, RFE, and XGB were shown to be the best of the implemented models. This is a realistic outcome because the best models (e.g., ANN) is known to be successful at detecting underlying patterns and significantly improves classification performance in a variety of classification tasks (e.g., malware detection [53], chatbot intent prediction [54]). We believe that collecting more qualified data will boost performance even further.

## 4. Discussion

Other than the performance of the ML models, we also investigated the best and worst features (i.e., molecular descriptors) selected by the RFE algorithm, as shown in Table 8. The estimated best pertinent and promising features from a ranking of features are assigned rank 1 [55,56] as shown in the table, so greater values of the rank imply worse features. All molecular descriptors in the PaDEL are grouped into some modules; for example, the molecular descriptor ‘nAcid’ belongs to the ‘acidic group count’ module as shown in the upper left corner of the table.

**Figure 3:**
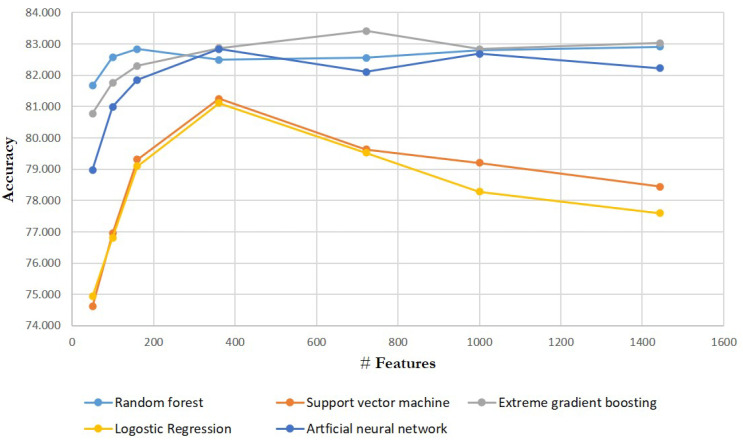
All ML models’ test set accuracies.

As we observed, when the number of descriptor values (i.e., selected molecular descriptor values) was 361 molecular descriptors, as shown in Figure 3, all models that were implemented in this research achieved a comparable performance of an accuracy above 81%, with the majority of the selected molecular descriptors coming from the ‘Autocorrelation module’. The ‘Autocorrelation’ module generates atom type autocorrelation descriptor values, and the autocorrelation descriptors are the molecular descriptors encoding both molecular structure and physico-chemical properties of a molecule [57,58,59,60] and also numerical properties assigned and attributed to atoms [59,61]. These descriptors are calculated by Moreau–Broto (ATS), Moran (MATS), and Geary (GATS) algorithms from lag 1 to lag 8 for four different weighting schemes [60,61,62]. The descriptors from the aforementioned module describe how a considered property is distributed in the topological molecular structure, and have a crucial influence on the antimalarial activity prediction [9]. This investigation is consistent with the previous studies of [59,63,64,65,66,67] which discussed the influence of such descriptors on antimalarial activity prediction towards the formation of drugs. It should be noted that the least relevant and worst descriptors come from ‘Atom type electrotopological state’ module, and it does not mean that these descriptors are detrimental to the performance or outcome. This precisely implies that the descriptors from the ‘Atom type electrotopological state’ contributed the least to the model compared to the others, so, due to this, it is reasonable to conclude that they have less influence on the discovery and development on antimalarial drugs.

We observed that, when the number of descriptor values (i.e., selected molecular descriptor values) was 361 molecular descriptors, as shown in Figure 3, all models that were implemented in this research achieved a comparable performance of an accuracy above 81%, with the majority of the selected molecular descriptors coming from the ‘Autocorrelation module’. In accordance with this, such small number of features may be prioritized for more expensive in-vitro antimalarial bioactivity screening and testing. This would result in a contribution of assisting the pharmaceutical chemists during the screening and formulation of a novel anti-malaria drug against Plasmodium falciparum by selecting and taking into account only the few and most promising and potential chemical features (i.e., molecular descriptors) from a pool of a majority of features.

It is worth noting that, in Table 9, the work of Egieyeh et al. reported the slightly higher accuracy compared to ours. This is due to the fact that the amount of data with regard to the number features was genuinely modest. Furthermore, we employed the same test dataset for all Implemented ML models, including the SVM used by Samuel Egieyeh, Although its performance was not superior as compared to the other deployed models in this research.

## 5. Conclusions

In this study, we used machine learning techniques to build various antimalarial predictive models that predict the bioactivity class of a drug against Plasmodium falciparum parasite. To address this antimalaria drug prediction problem, we employed the PaDEL, a well-known cheminformatics tool to extract the descriptor values following by the preprocessing. Experiments on molecular descriptor values of antimalaria drug chemical compounds retrieved from our collected data compounds revealed that the ANN and XGB models outperformed the other deployed ML models. In particular, XGB had the best recall 0.81 of the ‘active’ label and F1 score of 0.83 followed by ANN with recall of the ‘active’ and F1-score of 0.79 and 0.80, respectively. This implies that the XGB and ANN find about 81% and 79%, respectively, of new anti-malaria drug formation, both without losing too much precision. We believe that this research will assist in the discovery and development of anti-malaria drugs. We will look into gathering and collecting additional data in the near future, as having adequate data is essential for developing better ML models.

## Figures and Tables

**Table 1:** The glimpse sample of unprocessed data.

Service	ChEMBL_synonyms_PubChem_SID	Canonical_Isomeric_SMILES (Sources: PubChem_ChEMBL_and_EMBL-EBI)	Label
ChEMBL_&_PubChem	CHEMBL219517	C1CSCN(C1=O)CCCNC2=C3C=CC(=CC3=NC=C2)Cl	0
	380797	CC(C1=CC=CC=C1)NC(=O)C2=CC=CC=C2N=CC3=C(C=CC4=CC=CC=C43)O	0
	591362	C1=CC=C(C(=C1)C(=O)NC2=NC(=CS2)C3=CC=CC=N3)Br	0
	465546	C[C@@]1(CC[C@@H]2[C@]3(CC[C@@H](C([C@@H]3CC[C@]2(C1)O)(C)C)O)C)C=C	0
	341638	CCN(CC)CCCCSC1=C2C=CC(=CC2=NC=C1)Cl	0
	SID_381881704	CC1CN(CC(O1)C)C(=O)C2=C(C3=CC=CC=C3S2)OCC4=CC(=C(C=C4)F)F	1
	381885288	CC1CN(CC(O1)C)C(=O)C2=C(C3=CC=CC=C3S2)Cl	1
	381885327	CC1CN(CC(O1)C)C(=O)C2=C(C3=C(S2)C=C(C=C3)F)Cl	1
	381886215	CC1CN(CC(O1)C)C(=O)C2=NC3=CC=CC=C3S2	0
	381886674	CC1CN(CC(O1)C)C(=O)C2=C(C3=CC=CC=C3S2)OCC4=CC=CC=C4	1
	381886749	CC1CN(CC(O1)C)C(=O)C2=C(C3=C(S2)C=C(C=C3)C)Cl	1

**Table 2:** Data statistics.

	All Labels	Label ‘Active’	Label ‘Inactive’
# of data	4794	2070	2724

**Table 3:** Parameter settings of ML models.

Model	Setting
Random Forest	Number of estimators = 100
(RF)	No limitation of depth
	Minimum samples for splitting = 2
Support Vector Machine	Kernel = Linear
(SVM)	C = 1.0
Extreme Gradient Boosting	Number of estimators = 100
(XGB)	Learning rate = 0.3
Logistic Regression	Penalty = *l2*
(LR)	C = 1e5
	Class weight = None
	Multi_class = auto
	# of hidden layers = 2
Artificial Neural Network	# of nodes of each hidden layer = 100
(ANN)	Activation function = Relu [51]
	Optimizer = Adam [52]
	learning_rate = 0.0001
	# of epochs = 50 with early stopping

**Table 4:** Averaged test set accuracy of ML models, where $F_{ALL}$ is the number of all features, and $F_{S}$ means the number of features selected using the RFE algorithm.

Model	$F_{\mathrm{ALL}}$ = 1444	$F_{S}$ = 1000	$F_{S}$ = 722	$F_{S}$ = 361	$F_{S}$ = 160	$F_{S}$ = 100
RF	0.8294	0.8280	0.8256	0.8250	0.8284	0.8258
SVM	0.7850	0.7920	0.7964	0.8126	0.7931	0.7695
XGB	0.8318	0.8283	0.8342	0.8287	0.8230	0.8177
LR	0.7795	0.7828	0.7952	0.8111	0.7910	0.7682
ANN	0.8223	0.8269	0.8210	0.8283	0.8185	0.8100

**Table 5:** Per-label averaged test set precision of ML models, where $F_{ALL}$ is the number of all features, $F_{S}$ means the number of features selected using the RFE algorithm, and ‘Active’ and ‘Inactive’ mean label 1 and 0, respectively.

Model	$F_{\mathrm{ALL}}$ = 1444		$F_{S}$ = 1000		$F_{S}$ = 722		$F_{S}$ = 361		$F_{S}$ = 160	
	Inactive	Active	Inactive	Active	Inactive	Active	Inactive	Active	Inactive	Active
RF	0.8053	0.8462	0.8400	0.8712	0.7703	0.8467	0.8015	0.8583	0.8456	0.8457
SVM	0.7651	0.7121	0.7925	0.7102	0.7986	0.7801	0.8090	0.7958	0.8063	0.7795
XGB	0.8262	0.8020	0.8403	0.8429	0.8259	0.8387	0.8582	0.8477	0.8223	0.8125
LR	0.7973	0.8033	0.8148	0.7512	0.7958	0.7641	0.7643	0.7085	0.7819	0.7845
ANN	0.8381	0.8019	0.8405	0.8090	0.8316	0.8071	0.8433	0.8094	0.8345	0.7970

**Table 6:** Per-label averaged test set recall of ML models, where $F_{ALL}$ is the number of all features, $F_{S}$ means the number of features selected using the RFE algorithm, and ‘Active’ and ‘Inactive’ mean label 1 and 0, respectively.

Model	$F_{\mathrm{ALL}}$ = 1444		$F_{S}$ = 1000		$F_{S}$ = 722		$F_{S}$ = 361		$F_{S}$ = 160	
	Inactive	Active	Inactive	Active	Inactive	Active	Inactive	Active	Inactive	Active
RF	0.9066	0.7008	0.9131	0.7713	0.9114	0.6427	0.9115	0.7032	0.8908	0.7862
SVM	0.7904	0.6812	0.7721	0.7343	0.8456	0.7198	0.8566	0.7343	0.8419	0.7343
XGB	0.8566	0.7633	0.8897	0.7778	0.8897	0.7536	0.8897	0.8068	0.8676	0.7536
LR	0.8676	0.7101	0.8088	0.7585	0.8309	0.7198	0.7868	0.6812	0.8566	0.6860
ANN	0.8521	0.7837	0.8589	0.7841	0.8598	0.7696	0.8578	0.7891	0.8494	0.7775

**Table 7:** Per-label averaged test set F1 score of ML models, where $F_{ALL}$ is the number of all features, $F_{S}$ means the number of features selected using the RFE algorithm, and ‘Active’ and ‘Inactive’ mean label 1 and 0, respectively.

Model	$F_{\mathrm{ALL}}$ = 1444		$F_{S}$ = 1000		$F_{S}$ = 722		$F_{S}$ = 361		$F_{S}$ = 160	
	Inactive	Active	Inactive	Active	Inactive	Active	Inactive	Active	Inactive	Active
RF	0.8529	0.7666	0.8750	0.8182	0.8349	0.7306	0.8529	0.7730	0.8676	0.8148
SVM	0.7776	0.6963	0.7821	0.7221	0.8214	0.7487	0.8321	0.7638	0.8237	0.7562
XGB	0.8412	0.7822	0.8643	0.8090	0.8566	0.7938	0.8736	0.8267	0.8444	0.7820
LR	0.8310	0.7538	0.8118	0.7548	0.8129	0.7413	0.7754	0.6946	0.8175	0.7320
ANN	0.8445	0.7918	0.8493	0.7959	0.8452	0.7874	0.8501	0.7984	0.8417	0.7868

**Table 8:** Top best and worst features selected by the RFE algorithm when $F_{S}$ = 361.

Top50 Best Features			Top50 Worst Features		
**Module**	**Name**	**Rank**	**Module**	**Name**	**Rank**
Acidic group count	nAcid	1		nHmisc	1030
Atom count	nN	1		nsLi	1032
	nO	1		nssBe	1034
	nP	1		nssssBem	1036
Autocorrelation	ATS2m	1	Atom type electrotopological state	nsBH2	1038
	ATS4m	1		nssBH	1040
	ATS3v	1		nsssB	1042
	ATS4v	1		nssssBm	1044
	ATS3e	1		nssNH2p	1076
	ATS4e	1		nssAsH	1065
	ATS7e	1		nsssAs	1066
	ATS8e	1		SssBH	1047
	ATS3p	1		SddsN	1074
	ATS4p	1		SssAsH	1070
	ATS3i	1		SsssAs	1057
	ATS7i	1		SdsssAs	1058
	ATS8i	1		SsssssAs	1068
	ATS3s	1		SdSe	1043
	ATS5s	1		SssSe	1060
	AATS6v	1		SaaSe	1059
	AATS8e	1		SssSnH2	1035
	AATS6p	1		SsssSnH	1053
	AATS4i	1		SssPbH2	1083
	AATS6i	1		SsssPbH	1084
	AATS1s	1		minsBH2	1073
	AATS2s	1		minssBH	1069
	AATS5s	1		minssSiH2	1077
	AATS7s	1		minsssSiH	1075
	AATS8s	1		minssssSi	1080
	ATSC7c	1		minsPH2	1082
	ATSC8c	1		minssPH	1081
	ATSC3v	1		minddsP	1072
	ATSC4v	1		minsssssP	1071
	ATSC6v	1		minsGeH3	1051
	ATSC7v	1		minssGeH2	1052
	ATSC1e	1		minsssAs	1050
	ATSC2e	1		mindsssAs	1049
	ATSC3e	1		minddsAs	1048
	ATSC4e	1		minssSe	1046
	ATSC5e	1		minaaSe	1056
	ATSC6e	1		mindssSe	1055
	ATSC0p	1		minssssssSe	1054
	ATSC5p	1		minddssSe	1045
	ATSC6p	1		minsSnH3	1041
	ATSC8p	1		minssSnH2	1033
	ATSC1i	1		minsssSnH	1031
	ATSC4i	1		minsPbH3	1067
	ATSC7i	1		maxsBH2	1078
	ATSC8i	1		maxddsN	1037
	ATSC6s	1		maxaaS	1079

**Table 9:** Summary of comparison with previous studies.

	Samuel Egieyeh et al. [6]	Danishuddin, G et al. [9]	Our Work
Total # of data	1155	4750	4794
Total # of features	76	98	1444
Feature generation tool	RDKit	PaDEL	PaDEL
Feature selection	Feature Elimination	RFE	RFE, Kbest
Best model	SVM	SVM & XGBoost	ANN & XGB
Best accuracy (%)	85.93	∼85.00	∼83.00

## Data Availability

The data presented in this study are openly available in the website at http://sites.google.com/view/medardemswahili/ (accessed on 8 August 2021).

## Abbreviations

The following abbreviations are used in this manuscript:

WHO	World Health Organization
HTS	High-Throughput Screening
ACT	Artemisinin-based Combination Treatment
CQ	Chloroquine
APIs	Active Pharmaceutical Ingredients
QSAR	Quantitative Structure–Activity relationships
QSPR	Quantitative Structure–Property relationships
RFE	Recursive Feature Elimination
SMILES	Simplified Molecular-Input Line-Entry System
ANN	Artificial Neural Network
SVM	Support Vector Machine
RF	Random Forest
XGB	Extreme Gradient Boost
LR	Logistic Regression
SIDs	Substance Identifier
CIDs	Compound Identifier
InChIs	International Chemical Identifier
InChIKeys	International Chemical Identifier Keys
IUPAC	International Union of Pure and Applied Chemistry
SMO	Sequential Minimization Optimization
BLR	Binary Logistic Regression
